# Mapping protein–protein interactions by mass spectrometry

**DOI:** 10.1002/mas.21887

**Published:** 2024-05-14

**Authors:** Xiaonan Liu, Lawrence Abad, Lopamudra Chatterjee, Ileana M. Cristea, Markku Varjosalo

**Affiliations:** ^1^ Department of Physiology, Faculty of Medical Sciences in Katowice Medical University of Silesia in Katowice Katowice Poland; ^2^ Institute of Biotechnology, HiLIFE Helsinki Institute of Life Science University of Helsinki Helsinki Finland; ^3^ iCAN Digital Precision Cancer Medicine Flagship University of Helsinki Helsinki Finland; ^4^ Department of Molecular Biology Princeton University Princeton New Jersey USA

**Keywords:** interactomics, mass spectrometry, protein‐protein interactions, proteomics

## Abstract

Protein–protein interactions (PPIs) are essential for numerous biological activities, including signal transduction, transcription control, and metabolism. They play a pivotal role in the organization and function of the proteome, and their perturbation is associated with various diseases, such as cancer, neurodegeneration, and infectious diseases. Recent advances in mass spectrometry (MS)‐based protein interactomics have significantly expanded our understanding of the PPIs in cells, with techniques that continue to improve in terms of sensitivity, and specificity providing new opportunities for the study of PPIs in diverse biological systems. These techniques differ depending on the type of interaction being studied, with each approach having its set of advantages, disadvantages, and applicability. This review highlights recent advances in enrichment methodologies for interactomes before MS analysis and compares their unique features and specifications. It emphasizes prospects for further improvement and their potential applications in advancing our knowledge of PPIs in various biological contexts.

**Abbreviations**
AP: affinity purification
BiFC: bimolecular fluorescence complementation
BioID: proximity labeling‐dependent biotinylation identification
BRET: bioluminescence resonance energy transfer
CF: cofractionation
DDA: data‐dependent acquisition
DE: dynamic exclusion
DIA: data‐independent acquisition
FRET: fluorescence resonance energy transfer
GO: gene ontology
GOI: gene of interest
HCI: high confidence interaction
HRP: Horse radish peroxidase
IMAC: immobilized metal affinity chromatography
I‐PISA: Ion‐Based Proteome‐Integrated Solubility Alteration
MAC‐tag: Multiple Approaches Combined (MAC)‐tag
MS: mass spectrometry
PL: proximity labeling
POI: protein of interest
PPI: protein‐protein interaction
SEC: size‐exclusion
TPCA: Thermal Proximity Coaggregation
XL: cross‐linking

## 1 INTRODUCTION

Proteins are essential macromolecules that participate in a variety of biological processes such as signal transduction, transcription regulation, immunological defense, electron transfer, and metabolism. Over 80% of proteins interact with other proteins to accomplish their biological function or activity, rather than functioning alone (Berggård et al., 2007). These protein–protein interactions (PPIs) form the foundation of the protein interactome, a complex network that is critical for comprehending the function and regulation of biological networks. PPIs are typically categorized based on their stability and functional context. Stable interactions typically involve proteins that assemble into complexes with a long‐standing association, serving as the cellular infrastructure and machinery that are fundamental for processes such as DNA replication, RNA transcription, and the assembly of cytoskeletal structures. These protein complexes are frequently formed by precise folding and assembly processes that guarantee correct function and localization within the cellular environment. In contrast, transient interactions allow for swift assembly and disassembly of signaling complexes, facilitating a rapid and reversible response to stimuli. Moreover, the cellular milieu presents an exceedingly crowded environment. The high concentration of cellular proteins fosters a condition where even weakly interacting or transient contact may exhibit functionally relevant associations due to their proximal presence. For the systematic examination of PPIs, a variety of experimental techniques, including two‐hybrid systems, protein pull‐downs, protein chip technology, computational modeling, and various mass spectrometry (MS) based approaches, have been developed. With the enhanced sensitivity of MS and the use of bioinformatics tools for interactome data processing, protein pull‐down assay coupled with MS for PPI identification is the most sensitive method to investigate PPIs. However, in the context of mapping PPIs, particularly through techniques that involve cell lysis, only a fraction of PPIs with sufficient stability and appropriate kinetic parameters will remain intact for detection outside of the native cellular environment. This selective preservation underscores the importance of understanding the biochemical underpinnings of PPI stability and interaction dynamics. We have seen enormous progress in the field of PPI mapping by novel proteomics‐based approaches in the last decade, largely fueled by advancements in high‐throughput screening and large‐scale interaction studies enabled by the latest generation of MS instruments. These advancements have facilitated the systematic identification of PPIs on an exceptional level, greatly enhancing our comprehensive understanding of cellular functions. Several novel approaches for identifying and characterizing PPIs have been published and are beginning to rival affinity purification MS (AP‐MS) with the current generation of MS instruments and readily available analysis software streamlining PPI identification. This review discusses the current state‐of‐the‐art, as well as novel methodologies and their systemic application in the identification and characterization of PPIs within mammalian cells, highlighting the transformative impact of high‐throughput techniques in unveiling the complex network of protein interactions.

## 2 AP

AP‐MS is one of the most widely used approaches for systematically mapping protein interactions on a large scale. In this approach, the protein of interest (POI) is fused with an affinity tag and expressed in the cells of interest during culture. Following mild, nondenaturing cell lysis, the POI with associated proteins is collected using an affinity resin that recognizes the affinity tag. Thorough washing removes nonspecifically bound proteins, and elution of the POI (bait) and its interacting proteins (preys) is dependent on the particular affinity approach used but is often achieved via pH change, competing molecules, or a specific protease recognizing the tag.

**Figure 1:**
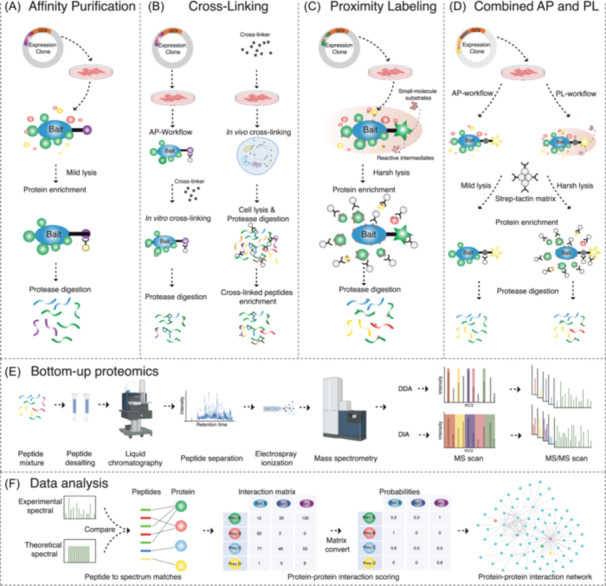
MS‐based approaches to studying interactomes. (A) Affinity tags are used in affinity purification (AP). Protein of interest (POI) that has been affinity tagged can be produced transiently or stably in selected cell lines. Subsequently, matrix conjugated to antiaffinity tag antibodies are added so that the tag fused POI and its interactors can be selectively enriched. (B) Cross‐linking can be done in vitro after purifying protein complexes or in vivo with intact cells. The cross‐linked proteins are digested to produce cross‐linked peptides, which are then enriched before mass spectrometry (MS) analysis. (C) The proximity labeling (PL) procedure. An enzyme is genetically fused to the POI and expressed in the cell line of choice. In vivo labeling is accomplished by introducing substrate into the cells, which converts these molecules into reactive intermediates for PL. Labelled proteins can be enriched. (D) Combination of AP and PL. Depending on the culture state and lysis buffer combination, MAC‐tagged POI can be utilized for both AP and PL procedures. The same matrix is used to enrich protein complexes. (E) In bottom‐up proteomics, peptides derived from proteolytic digestion are first desalted to remove salts that can interfere with subsequent analyses. The cleaned peptide mixture is then loaded onto an liquid chromatography column for liquid chromatography, followed by electrospray ionization. Ionized peptides are analyzed using two primary MS strategies: data‐dependent acquisition (DDA), where ions are scanned and the most abundant peptides are chosen for MS/MS scans, and data‐independent acquisition (DIA), where all peptides within a set mass range are systematically fragmented. These approaches yield tandem mass spectra for peptide identification and inference of associated proteins. (F) Data analysis begins with the comparison of experimental spectra against a theoretical database to establish peptide–protein matches, which then form an interaction matrix. Interaction probabilities are statistically scored to recognize high‐confidence interactions (HCIs). These HCIs, extracted from filtering processes, are used to construct a protein–protein interaction network, elucidating the intricate web of protein interactions within the cellular environment. [Color figure can be viewed at wileyonlinelibrary.com]

The versatility of AP‐MS (Figure 1A) is expanded by the diverse range of affinity tags available, from small peptide sequences to larger protein domains, which can be positioned at the N‐terminus, C‐terminus, or even the middle of the POI sequence. The precise positioning of these tags is crucial and needs experimental fine‐tuning to maintain the most natural interactions during the purification process. These additional sequence fragments introduce specific properties to the POI. Therefore, an optimal tag for large scale PPI investigations should be compact (small in size) to avoid steric hindrance and potential alterations in the function or location of the POI, yet robust enough to withstand the purification process without dissociating from its binding partners.

Through the application of this approach, the first comprehensive human protein interactome was elucidated (Ewing et al., 2007), demonstrating the effectiveness of AP‐MS in uncovering the complex network of interactions on a systemic level. Advancements in MS technology are enhancing the accuracy of PPI detection, allowing for deeper understanding of the intricate molecular interactions within cells and the complex protein networks that support cellular structure and function.

### 2.1 Protein affinity tags

Very few proteins are used as affinity tags (Table 1) in interactomics due to their large size and complex structure. Large proteins are more prone to suffer protein misfolding (Chiti & Dobson, 2017), hence they are less likely to be used as tags than peptides, perhaps interfering with the proper folding or function of the POI. Furthermore, their size and complexity can lead to unspecific binding, in which the tag adheres to proteins other than the intended interaction partners, complicating the detection of genuine PPIs. Furthermore, these large proteins present practical challenges in cloning and expression. These issues demand a delicate balance between tag size, convenience of use, and the accuracy of captured interactors.

**Table 1:** Affinity tags employed in protein interaction studies and their general use conditions.

Affinity purification tag	Size (amino acid)	Origin	Variation in tags	Application	Matrix	Variation in matrix/ligands	Elution
GFP	220	*Aequorea Victoria*	eGFP	Detection, purification, FACS	Anti‐fluorescent antibodies, nanobodies	Anti‐fluorescent nanobodies	SDS sample buffer
Halo‐Tag7	312	Rhodococcus rhodochrous	Halo‐Tag7	Purification, increased solubility	HaloLink™ Resin	HaloTag® Biotin Ligand, HaloTag® PEG‐Biotin Ligand	HaloTag® Buffer, TEV Protease
PIF6	22	*Arabidopsis thaliana*	PhyB‐PIF3	Detection, purification	NeutrAvidin	N/A	Biotin
FLAG‐tag	8 (DYKDDDK)	Synthetic peptide	3X Flag‐tag	Detection, protein purification, immunoprecipitation	mAb,	Anti‐FLAG nanobody, Anti‐FLAG magnetic beads	Low pH, EDTA, FLAG peptide
HA‐tag	31	Human influenza virus	3X HA‐tag	Detection, protein purification, immunoprecipitation	mAb	N/A	Low pH, HA peptide
Strep‐tag	8–9 (WSHPQFEK) or (AWAHPQPGG)	Synthetic peptide	Strep‐tag II	Detection, protein purification,immobilization	Streptactin	Streptavidin, Streptactin magnetic beads	Biotin
c‐Myc/Myc	11 (CEQKLISEEDL)	Human c‐Myc	N/A	Detection, protein purification, immunoprecipitation	mAb	Anti‐Myc nanobody, Anti‐c‐Myc magnetic beads	Low pH
His‐tag	6 (HHHHHH)	Synthetic peptide	His tag with glycine‐serine linker peptide	Detection, protein purification, immobilization	Divalent metal	supermagnetic nanoparticles	Imidazole, low pH
SpyCatcher‐SpyTag	13 (AHIVMVDAYKPTK)	Streptococcus pyogenes	SpyDock (Spy&GO)	Detection, purification	Ni‐NTA	N/A	Low pH
ALFA‐tag	13 (AAALEHHHHHH)	Synthetic peptide	Different affinities of ALFA (e.g., NbALFA, NbALFAPE)	Detection, purification	ALFA Selector Resin	ALFA‐tag specific nanobodies	Competitive elution with ALFA peptide, low pH

#### 2.1.1 Fluorescent proteins

Fluorescent tags have the capacity to emit light once they are exposed to ultraviolet (UV) light. The subcellular location of fluorescent‐labeled POI can be determined by observing them under a microscope. Fluorescent tags are also helpful in facilitating fluorescence‐activated cell sorting to differentiate transgene‐positive cells from negative clones. For interaction studies, fluorescent‐tagged proteins can be concentrated using an affinity matrix with specific antibodies on. Green fluorescent protein (GFP) from *Aequorea victoria*, Yellow Fluorescent Protein (YFP), and the mFruits family (e.g., mCherry) are commonly used fluorescent proteins for tagging due to their wide availability in the market, resistance to fading, and minimal harm to cells (Cong et al., 2022).

The GFP‐tag, in particular, has been extensively used (Cristea et al., 2005) and has been combined with high‐throughput cloning techniques for large‐scale interactomes studies (Trinkle‐Mulcahy et al., 2008). For instance, the quantitative BAC‐GFP interactomics (QUBIC) technique (Hubner et al., 2010), labels genes of interest (GOI) with GFP using the bacterial artificial chromosome (BAC) transgenic technology. This QUBIC approach was then applied to study the human interactome. Researchers analyzed 1125 GFP‐fused POIs that were expressed in HeLa cell lines and purified using an immobilized GFP antibody (Hein et al., 2015). The study reported specific interactions for selected POIs, estimation of interaction stoichiometry, and measurement of interactor cellular abundances.

Nanobodies with a single antigen‐binding domain have recently revolutionized antibody‐based protein purification due to their exceptional stability and selectivity. There are several antifluorescent protein nanobodies with high affinity and specificity (Cong et al., 2022). Even for POI expressed at very low levels, nanobodies can be exceedingly rapid and efficient in protein pull‐down. Moreover, compared to standard antibody‐based matrix, nanobody‐based matrixes exhibit exceptional stability, selectivity, and high affinity for fluorescent proteins tagged POI. Their efficiency and specificity in AP assays have made them invaluable in protein purification and interaction studies. Other innovations such as lysine‐free nanobody has been developed that limit the number of tryptic peptides released from the affinity matrix during on‐bead digestion to reduce the complexity of mass spectrometry analysis (Shi et al., 2015).

Expanding upon these methodologies, the OpenCell approach (Cho et al., 2022) combines CRISPR‐mediated genome editing with live cell fluorescent labeling to systematically explore the localization and interactions of human POIs. By tagging 1310 POIs in HEK293 cell lines with fluorescent markers and analyzing with confocal microscopy and MS, OpenCell provides a comprehensive proteomic map of human cellular architecture (Cho et al., 2022). This data‐driven study highlights functional communities across the proteome, underlining the distinct interaction and localization properties of RNA‐binding proteins. Unlike standard tagging methods, OpenCell uses endogenous tagging to maintain endogenous protein expression levels and enables real‐time observation of protein dynamics in their natural cellular context. This improves our understanding of protein localization, movement, and interactions, highlighting the importance of fluorescent tags in current proteomics for capturing the dynamic nature of proteins in living cells.

#### 2.1.2 Haloalkane dehalogenase (HLD) enzyme (HaloTag)

The HaloTag system (Los et al., 2008) is based on an engineered HLD from *Rhodococcus rhodochrous*, which can be used for versatile protein tagging and functional analysis. The HaloTag can be covalently linked to any synthetic ligand such as a biotin or epitope tag, which has been modified to contain a reactive chloroalkane linker. The flexibility of the HaloTag system is further enhanced by the availability of various ligands, each with different functionalities thus only a single genetic construct is required for studying different aspects of the POI. For example, HaloTag was used to investigate the subcellular distribution and function of the Sin3 histone deacetylase complex (Adams et al., 2020; Banks et al., 2018). By using a fluorescent HaloTag ligand, the localization of HaloTag fused POIs can be visually tracked, offering insights into protein trafficking, organelle organization, and interaction networks within live cells. For comprehensively mapping interactions, protein complexes can be rapidly concentrated using an affinity HaloTag ligand (Adams et al., 2020; Banks et al., 2018).

#### 2.1.3 Phytochrome interacting factor (PIF/PhyB system)

An optogenetics‐based light‐controlled AP method (Hörner et al., 2019) was developed utilizing the light‐dependent PPIs properties between phytochrome B (PhyB) and phytochrome interacting factor 6 (PIF6) from *Arabidopsis thaliana*. PhyB transitions to its Pfr conformational state (PhyB far‐red absorption state) in response to 660 nm red light, where it interacts with PIF6 with nanomolar affinity (Khanna et al., 2004). The conformational shift of PhyB to the Pr state (PhyB red absorption state) at 740 nm far‐red light inhibits the interaction with PIF6. As a result, the truncated variant of PIF6 can serve as an affinity tag fused with the POI. Biotinylated PhyB is immobilized on NeutrAvidin (N)‐functionalized agarose beads as binding matrix. After cell lysis, the fusion POI can bind to PhyB via its PIF tag under 660 nm light and eluted by switching illumination to 740 nm. Researchers were able to isolate PIF6‐tagged ZAP70 and its interaction partners from both Jurkat and P116‐derived cell lines using this PhyB/PIF system (Hörner et al., 2019). In comparison to other AP techniques, using light to modulate protein complex elution is a gentle condition that effectively lowers the detection of false positive PPIs.

### 2.2 Peptide affinity tags

Small‐size tags, as opposed to protein affinity tags, have the advantage of reduced impact on the structure, activity, and properties of the recombinant protein, and are commonly favored in interactomics studies. A series of short peptide‐based tags has been developed (Table 1).

#### 2.2.1 FLAG‐tag

The FLAG‐tag is a hydrophilic octapeptide (DYKDDDDK) that can be recognized by specific antibodies. There are several monoclonal anti‐FLAG antibodies, including M1, M2, and M5, each with a unique recognition and binding profile (Brizzard et al., 1994; Slootstra et al., 1997). Several companies also sell polyclonal antibodies with stronger affinity than these monoclonals.

FLAG‐tagged protein complexes can be eluted from the affinity beads by using competitive displacement (e.g., 3× FLAG peptide) (Brizzard et al., 1994) or a low pH buffer (e.g., 1 M arginine at pH 3.5–4.4) (Futatsumori‐Sugai et al., 2009). FLAG tag does not react with the chemicals used in standard AP‐MS workflows and offers efficient elution aimed for in proteomics studies. It is widely used in both academic research and pharmaceutical industries. In the first reported large‐scale human interactome study (Ewing et al., 2007), 338 FLAG‐tagged baits were profiled by an AP‐MS workflow. However, previous studies (Guo et al., 2021) have shown that when the FLAG‐tag is fused with specific secreted POI in the cell, the tyrosine residue in the FLAG‐tag might be sulfated, impairing antibody recognition of the FLAG‐tag (Guo et al., 2021).

A recent study (Ang et al., 2022) proposed a new approach utilizing Field Asymmetric Ion Mobility Spectrometry (FAIMS) (Swearingen & Moritz, 2012) for FLAG‐tagged protein complex detection. This method significantly increases proteome coverage by filtering out the highly abundant FLAG peptides that are added during the elution process, which can dominate the MS analysis and mask less abundant peptides detection from the target proteins. By applying FAIMS with specific compensation voltages, researchers demonstrated not only an increase in protein identification but also a deeper biological insights of interacting proteins, offering new insights without requiring additional sample preparation (Ang et al., 2022). This innovation marks a big step forward in the application of FLAG‐tagging for protein complex analysis.

#### 2.2.2 Strep‐tag

The Strep‐tag was first discovered from a random peptide library as an eight‐amino acid peptide (WRHPQFGG) that binds exclusively to streptavidin (Pähler et al., 1987; Schmidt & Skerra, 1993). The Strep‐tag/streptavidin system has been optimized over time, with the development of the optimized Strep‐tag II (WSHPQFEK) (Schmidt et al., 1996), allowing for greater flexibility in the choice of the attachment site and engineered streptavidin variant, known as Strep‐Tactin. The binding affinity of the Strep‐tag II may be increased by 100 times using Strep‐Tactin (Schmidt et al., 2013). The Twin‐Strep‐tag (WSHPQFEKGGGSGGGSGGSAWSHPQFEK) originally known as Strep‐tag III (Junttila et al., 2005, Schmidt et al., 2013), was invented to improve the Strep‐tag II system in challenging situations where require higher binding affinity and stability, while maintaining the reversible and gentle elution conditions of protein complex purification. The most recent improvement of the Strep‐tag/streptavidin involved the creation of Strep‐Tactin XT (Yeliseev et al., 2017). The Strep‐tag II binds with a low nanomolar binding affinity, while the Twin‐Strep‐tag binds with a low picomolar binding affinity (Yeliseev et al., 2017).

Mammalian cells have four known endogenously biotinylated proteins (Chandler & Ballard, 1986). To avoid binding of endogenous biotinylated proteins during purification, avidin is added to the lysate to mask these proteins. This process may produce a precipitate, which must be removed by centrifugation before proceeding to the chromatography steps (Moosmeier et al., 2010; Schmidt & Skerra, 2007). Because of the strong binding affinity, the Strep‐tagged protein may be purified efficiently in a fast single‐step process. Furthermore, the Strep‐tag has several advantages over other short peptide affinity tags. These benefits include the fact that it is biologically inert, proteolytically stable, typically does not impair POI folding or localization, and does not cause protein aggregation. Strep‐tag is thus particularly useful for studying functional proteins or protein complexes, and is suitable also for structural proteomics studies (Laulumaa et al., 2024).

#### 2.2.3 Polyhistidine (His6)

The hexahistidine (His6) affinity tag is another widely used tag for AP workflow. A His‐tagged protein, along with its interacting partners, is purified using immobilized metal affinity chromatography (IMAC), where divalent cations (Co, Cu, Ni, or Zn) adhere to a solid matrix (Schmidt & Skerra, 2007; Spriestersbach et al., 2015). The protein complex of His‐tagged POI can be eluted by using a high concentration of imidazole, a low pH, or an excess of strong chelators. However, since naturally occurring histidine‐rich proteins are found in a variety of biological systems (Poon et al., 2011), their affinity for the IMAC raises the possibility of significant background interactions in AP studies using His‐tags. Addressing this challenge, recent advancements include the development of a novel AP method to purify His‐tagged POI in tip format (Liu et al., 2020). These spin‐tip micro monolithic columns with the IMAC (m‐IMAC) were prepared by photoinitiated copolymerization in the pipette tips. The usage of the m‐IMAC greatly streamlines the operation while simultaneously increasing purifying efficiency.

#### 2.2.4 SpyCatcher‐SpyTag system

The SpyCatcher‐SpyTag system (Liu et al., 2020) is based on a modified immunoglobulin‐like domain from *Streptococcus pyogenes* surface protein, which recognizes a cognate 13‐amino‐acid peptide (SpyTag). SpyCatcher is characterized by its reactive lysine and catalytic glutamate, while SpyTag comprises reactive aspartate. The two components have a strong affinity for each other (0.2 µM) and can form a covalently bound complex (Keeble et al., 2017). The SpyTag can be genetically fused to a POI, whereas SpyCatcher can be fused to epitope or purification tags, thus adding extra steps to the cloning process. Addressing this, the Spy&GO system was developed by stepwise engineering the SpyCatcher protein to form SpyDock matrix (Khairil Anuar et al., 2019), which can recognize and bind SpyTag directly. SpyTag has been used extensively in vaccine development to form covalently stabilized multiprotein complexes and to investigate the activities of outer membrane proteins (Tan et al., 2021; Thrane et al., 2016).

#### 2.2.5 ALFA‐tag system

ALFA‐tag (SRLEEELRRRLTE) (Götzke et al., 2019), was developed based on an artificial peptide known to have a stable α‐helix structure in solution (Petukhov et al., 2009). The ALFA‐tag is distinguished by its hydrophilic nature and balanced charge distribution, ensuring little interference with the biological function or localization of the tagged POI. A set of highly versatile single‐domain antibodies, NbALFA, NbALFA^PE^, and NbALFA^CE^ can bind ALFA‐tagged proteins with extraordinary specificity regardless of the position of the ALFA‐tag on the POI (Kilisch et al., 2021). NbALFA has a high affinity (Kd ~25 pM) for the ALFA‐tag, making it appropriate for applications that need strong binding, like super‐resolution imaging and live‐cell protein monitoring (Kilisch et al., 2021). NbALFA^PE^ and NbALFA^CE^ can meet the requirement for reversible protein binding under physiological conditions (Kilisch et al., 2021), especially NbALFA^CE^ allowing effective protein elution at 4°C, widening the system's application to temperature‐sensitive proteins. This modular strategy allows tag‐antibody interactions to be tailored to specific experimental conditions, such as stable complex formation and gentle elution procedures that protect protein integrity. It is important to carefully select the suitable nanobody (NbALFA, NbALFA^PE^, or NbALFA^CE^) based on the specific application. This decision should consider aspects such as the requirement for a stable connection versus reversible binding.

#### 2.2.6 Dual‐tagging system

The tandem affinity purification (TAP) method (Puig et al., 2001; Rigaut et al., 1999) by combining two affinity tags was initially developed in yeast. The TAP tag allows for an initial affinity purification directed towards the outer tag. Protease cleavage releases the concentrated protein–prey complexes, which are subsequently isolated again through a second affinity purification method based on the inner tag and identified using mass spectrometry. This three‐step process enhances specificity and has proven most useful in the systematic identification of numerous multiprotein complexes in the yeast (Gavin et al., 2006; Krogan et al., 2006), but has shown inconsistency when applied to mammalian systems, occasionally resulting in low yields of protein complexes (Cox et al., 2002). To address these limitations, a refined dual‐tag system was developed (Giannone et al., 2007), enhancing specificity and efficiency by integrating two distinct affinity tags, regulated expression, and optimized purification protocols. For example, sodium dodecyl sulfate polyacrylamide gel electrophoresis fractionation free the dual‐tag system containing both Twin‐Strep (‐tag and HA‐tag was utilized to characterize the PPIs networks of several subsets protein phosphatases and protein kinases (Glatter et al., 2009; Varjosalo, Keskitalo, et al., 2013; Varjosalo, Sacco, et al., 2013). To increase the purity of target protein complexes for MS analysis, the cell lysate was first loaded onto Strep‐Tactin® matrix, and the elution was then incubated with anti‐HA agarose. However, the inherent complexity and time demands of this method have led to a transition towards more streamlined, single‐step affinity pull‐down approaches.

## 3 CROSS‐LINKING MS

Although AP‐MS experiments identify specific interactors/protein complexes for any given bait, the results neither differentiate the direct and indirect interactions between the bait protein and its interaction partners, nor provide detailed structural or topological information on protein complex assemblies. Therefore, cross‐linking MS (XL‐MS) has emerged as a powerful tool for deciphering the structures of protein complexes by direct observation of subcomplex interfaces at the peptide level (Lee & O'Reilly, 2023; Yu & Huang, 2018). XL‐MS (Figure 1B) employs cross‐linkers to covalently bond proteins. XL‐MS allows for the identification of cross‐linked peptides and the residues in close‐proximity within the protein complex. The proximity determination relies on the specified length of the spacer‐arm of the crosslinker to accurately identify interaction sites within the complex. Another significant advantage of XL‐MS, particularly when applied in vivo, is its capacity to access PPIs that are challenging to study using AP‐MS. This includes interactions involving proteins that are not easily solubilized or those with high dissociation rates, which might evade detection in traditional AP‐MS setups.

However, the process of isolating low‐abundance and heterogeneous cross‐link products from complex peptide mixtures presents a significant challenge, particularly for proteome‐wide analysis. As a result, the cross‐linking reaction is often performed in vitro after affinity purification to enhance the specificity of XL‐MS (Figure 1B). A notable application of this approach was the extraction of the GFP‐tagged Beclin1 protein complex from transgenic mouse liver using non‐cross‐linkable nanobody‐conjugated magnetic beads (Shi et al., 2015). The protein complex was then cross‐linked on beads with disuccinimidyl suberate (DSS) for MS analysis to model its architecture in its native state. The numbers of crosslinks identified on these native GFP‐tagged complexes can delineate structural details of the complexes being analysed.

The XL‐MS has evolved significantly, offering diverse workflows that extend beyond the crosslinking post‐AP. Recent methodologies include performing crosslinking in lysates or applying in situ crosslinking before AP (Lee & O'Reilly, 2023). These approaches enhance the detection of transient or weak PPIs by stabilizing complexes before their isolation. For example, an innovative method combines AP and cross‐linking with photo‐reactive amino acids to explore interactions of protein kinase D2 (PKD2) (Häupl et al., 2017). This technique started with culturing HeLa cells and supplementing them with photo‐reactive amino acids, specifically photo‐methionine and photo‐leucine to substitute their natural counterparts and to be incorporated into the synthesized proteins. Subsequently, immobilized GST‐tagged PKD2 protein was exposed to the HeLa cell lysate enriched with these photo‐reactive amino acids. The key step involves using UV‐A irradiation to induce cross‐linking exclusively among proteins that have integrated these special amino acids, thus preserving protein interactions occurring at the moment of irradiation.

Another innovative approach to investigate the structures of SARS‐CoV‐2 nonstructural protein (NSP) (Slavin et al., 2021), gene of interest (NSP1 and NSP2) were tagged with a Strep‐tag and expressed in human cells. These cells expressing the viral proteins were treated with a membrane‐permeable cross‐linking reagent (either DSS or formaldehyde), to facilitate the formation of covalent bonds between amino acids that are close together in the three‐dimensional structure. After cross‐linking, the viral proteins formed complexes were isolated through purification using Strep‐Tactin resin. This process facilitated the generation of structural models that shed light on the function of viral proteins, revealing potential protein–RNA interactions and pinpointing likely metal‐binding sites within NSP2. This approach underscores a novel pathway for delving into the structural intricacies of viral proteins directly within their native cellular milieu.

Additionally, innovative strategies such as APEX‐based PL combined with XL‐MS have been developed (Sun et al., 2022), further expanding XL‐MS capabilities to study the dynamic interactome within cells. Cells expressing the APEX2 fused POI were incubated with biotin‐phenol and hydrogen peroxide (H_2_O_2_) to initiate APEX labeling. After APEX labeling, cells were lysed, and the lysate was treated with the cross‐linking reagent DSS to form covalent bonds between amino acids. Biotinylated proteins were then purified using streptavidin beads, and cross‐linked protein complexes were enriched through this process. The workflow specifically applied to mitochondria and nuclei, identifying hundreds of interprotein cross‐links and providing insights into sub‐organelle interactomes with high specificity.

Advancements in XL‐MS have also extended its application to proteome‐wide analysis to offer a global view of the dynamics of protein complexes in the context of a cell. This is still an area of active workflow development and various efforts have been made to develop new XL reagents, enrichment strategies, and bioinformatics tools to enable system‐wide XL‐MS (in vivo XL‐MS) experiments (Figure 1B). In XL‐MS, peptide fractionation has become indispensable, given the XL‐MS technique generates four distinct types of cross‐links during its process: (i) cross‐links, (ii) mono‐links (dead‐end links), (iii) loop‐links, and (iv) unreacted peptides. Among these, cross‐linked and mono‐linked peptides are of particular interest because they provide valuable structural information, albeit being the least abundant. This variety complicates XL‐MS analysis by requiring the differentiation of informative peptides from a complex mix. Simplifying the sample to improve the detection of these key peptides is therefore essential.

For example, biotinylated cross‐linkers were developed to enable effective cross‐linked peptides enrichment (Tang & Bruce, 2010). After cross‐linking, biotinylated cross‐linked peptides are captured by avidin beads before MS analysis. Other cross‐linkers that efficiently fragment in gas‐phase fragmentation, to generate two major fragment ions corresponding to the component peptides have evolved to speed up and simplify the analysis of MS data (Liu et al., 2015, 2017).

The introduction of IMAC‐enrichable crosslinkers, such as PhoX (Steigenberger et al., 2019), has provided a more efficient option for peptide enrichment. The single molecule, PhoX, composes of two NHS‐ester groups which react with lysine residues in proteins and a phosphonic acid handle which allows for IMAC enrichment. Treatment with PhoX covalently binds lysine residues within or between proteins when they are in close proximity. After cross‐linking, proteins are digested into peptides, which then undergo Fe‐IMAC to enrich for PhoX‐modified peptides. The phosphonic acid group of PhoX has a strong affinity for IMAC matrix metal ions, enabling the selective enrichment of cross‐linked and mono‐linked peptides and reducing unmodified peptide background. The application of PhoX overcomes challenges related to the low stoichiometry of cross‐linking reactions and the complexity of peptide mixtures, significantly improving the detection of cross‐linked peptides in MS studies.

With these improvements, in vivo XL‐MS has been successfully employed to define cellular PPIs and complex assemblies in the nucleus, mitochondria, synaptic vesicles, mouse brain, mouse heart, and several cell lines (Piersimoni et al., 2022; Wang, Hu, et al., 2022). However, XL‐MS faces certain limitations, including the poor effectiveness (1%–5%) of cross‐linking reagents. This often results in a limited number of cross‐links, typically capturing only the top 20%–30% of highest expressed proteins (Low et al., 2021). Moreover, determining the optimum timing and dosage of cross‐linkers to use to preserve particular PPIs without adding additional nonspecific contaminants can be challenging. These unwanted elements can range from irrelevant proteins and environmental keratins to unintended cross‐linker reactions and residual chemicals from sample preparation, all of which can obscure the true biological interactions under study.

Overall, the field of XL‐MS is rapidly advancing, with a plethora of workflows being developed and refined to capture a wide array of PPIs and complex assemblies. Each approach offers unique advantages and is suited for specific research questions, underlining the importance of selecting the appropriate methodology based on the biological context and the nature of the interactions under investigation.

## 4 PL

A limitation of AP‐MS is the need for mild lysis conditions to maintain the protein complexes. Particularly, membrane proteins can be hard to extract under native lysis conditions due to the lysis buffer composition. Another limitation of AP‐based methods is the bias towards proteins that interact with high affinity and with slow kinetics of dissociation. Therefore, in recent years, several alternative enzyme‐catalyzed PL approaches have been developed in recent years as powerful complementary approaches to AP‐MS. In a PL system, the POI or subcellular compartment marker proteins are fused in frame to a promiscuous labeling enzyme that can transform an inert substrate into a reactive but transient intermediate, which then covalently modifies the adjacent proteins in a proximity‐dependent way. In most cases, the substrates are biotin or biotin‐derived small molecules. Therefore, those labeled proteins can be selectively enriched by protein purification using the Strep‐Tactin matrix before MS analysis (Figure 1C). Depending on the enzyme utilized (Table 2), these PL approaches can track the history of POI associations over time in living cells or capture a snapshot of associated proteins. Here, we focus on the applications of several PL‐based methods in proteomics studies and discuss advantages and limitations of the different methods.

**Table 2:** Overview on the characteristics of major proximity labeling tags methods.

Proximity labeling tag	Enzyme	Origin	Size (kDa)	Mutations	Substrate	Time of labeling	Labeling radius (nm)	Labeling residues	Application	Screen unknown interactions	Readout
**BioID**	Biotin protein ligase	*E.Coli*	35	R118G	Biotin (0.1‐50 μM)	16–18 h	~ 10	Lys	*In vitro* and *in vivo*	Yes	Mass spectrometry
**BioID2**	Biotin protein ligase	*A.Aeolicus*	26.4	R40G	Biotin (0.01‐ 12.5 μM)	16–18 h	~ 10‐15	Lys	*In vitro;in vivo (unknown)*	Yes	Mass spectrometry
**TurboID**	Biotin protein ligase	*E.Coli*	35	Q65P, I87V, R118S, E140K, Q141R, A146Δ, S150G, L151P, V160A, T192A, K194I, M209V, M241T, S263P, I305V	Biotin (50/500 μM)	10 min	~ 10	Lys	*In vitro* and *in vivo*	Yes	Mass spectrometry
**minTurboID**	Biotin protein ligase	*E.Coli*	28	N‐terminal aa1‐63 delated; Q65P, I87V, R118S, E140K, Q141R, A146Δ, S150G, L151P, V160A, T192A, K194I, M209V, I305V	Biotin (50/500 μM)	10 min	~ 10	Lys	*In vitro* and *in vivo*	Yes	Mass spectrometry
**2C‐BioID**	BioID and FKBP:FRB system	*E.Coli*	35	R118G	Biotin and ATP; Rapamycin	16–18 h	~ 10	Lys	*In vitro;in vivo (unknown)*	Yes	Mass spectrometry
**Split‐BioID**	Biotin protein ligase	*E.Coli*		Split BioID at aa140/141 or aa256/257	Biotin and ATP	16–18 h	~ 10	Lys	*In vitro;in vivo (unknown)*	NO	Mass spectrometry
**APEX**	Ascorbate peroxidase	*Pea* or *Soybean*	~ 28	K64D, W41F, E112K	H2O2 and biotin‐phenol	1 min	~ 20	electron‐rich amino acids such as Tyr, Trp, His and Cys	*In vitro* and *in vivo*	Yes	Mass spectrometry; Electron microscopy
**APEX2**	Ascorbate peroxidase	*Soybean*	28	K64D, W41F, E112K, A134P	H2O2 and biotin‐phenol	1 min	~ 20 (more active than APEX)	electron‐rich amino acids such as Tyr, Trp, His and Cys	*In vitro* and *in vivo*	Yes	Mass spectrometry; Electron microscopy
**Split‐APEX**	Ascorbate peroxidase	*Soybean*		Split APEX2 at aa200/201 TheN‐terminal fragment (aa 1–200; K22R, R24G, G50R, K61R, H62Y, I165L, N72S, P125L, I185V); The C‐terminal fragment (aa201–250)	H2O2 and biotin‐phenol	1 min	~ 25	electron‐rich amino acids such as Tyr, Trp, His and Cys	*In vitro* and *in vivo*	No	Mass spectrometry
**SPPLAT**	Horseradish peroxidase	*Horseradish*	38	HRP‐conjugated antibody	H2O2 and biotin‐phenol	5 min	10–200	Tyr	*In vitro*	Yes	Mass spectrometry
**BAR**	Horseradish peroxidase	*Horseradish*	38	HRP‐conjugated antibody	H2O2 and biotin‐phenol	10 min	Not specific measured >10	Tyr	*In vitro* (extend the use of HRP to tissue)	Yes	Mass spectrometry
**PUP‐IT**	Pup‐‐protein ligase	*C. glutamicum*	53	Codon‐optimized for mammalian cell expression	Pup(E) derivative peptides and ATP	24–36 h	Unknown (close contact)	Lys	*In vitro*	Yes	Mass spectrometry
**NEDDylator**	NEDD8‐conjugating enzyme Ubc12	*Human*	75	XIAP (aa1‐434) is fused via a flexible Gly‐Gly‐Ser‐Gly linker to the NEDD8 E2, Ubc12	His‐biotin‐tagged NEDD8		Unknown (close contact)	Lys	*In vitro*	Yes	Mass spectrometry
**airID**	Biotin protein ligase	*designed de novo*	37	Based on ancestral BirA, designed de novo	Biotin (50 μM)	6 h	Unknown (close contact)	Lys	*In vitro*	Yes	Mass spectrometry
**UltraID**	Biotin protein ligase	*A.Aeolicus*	19.7	Truncation variant of BioID2 (aa2‐171, R40G&L41P)	Biotin (50 μM)	10 min	Unknown (close contact)	Lys	*In vitro*	Yes	Mass spectrometry

### 4.1 Biotin ligases based PL

One of the earliest and most widely used PL techniques is PL‐dependent biotinylation identification (BioID)(Roux et al., 2012), which uses a mutant of the *Escherichia coli* biotin ligase (BirA) called BirA*(BirA R118G, BirA*) (Choi‐Rhee et al., 2004) fused to the POI. BirA* utilizes biotin to create highly reactive biotinoyl‐5‐AMP that can diffuse to biotinylate proteins within a radius of approximately 10 nm. The covalent addition of biotin allows the labeled proteins to be captured in a single‐step streptavidin purification. Since its introduction and initial application in 2012, BioID has made significant contributions to various proteomics studies (Eesmaa et al., 2021; Gawriyski et al., 2024; Göös et al., 2019, 2022; Heikkinen et al., 2017; Kinnunen et al., 2023; Paakkola et al., 2018; Roux et al., 2018; Yadav et al., 2017), including those involving insoluble, inaccessible, or low‐abundance structures such as chromatin (Lambert et al., 2015), centrosomes (Gupta et al., 2015), cell junctions (Van Itallie et al., 2013), the endoplasmic reticulum (Hoyer et al., 2018), signaling pathways (Zhou et al., 2015), and SARS‐CoV‐2 virus‐host protein interactions (Liu et al., 2021; Rees et al., 2015).

One major disadvantage of BioID is the size of the fused BirA* (~35 kDa), which can potentially affect the localization and/or function of the POI. For example, BioID tagging has been shown to restrict the passage of lamina‐associated polypeptide 2β (LAP2β) through nuclear pore complexes (NPCs) (Chojnowski et al., 2018). Additionally, BioID has very slow kinetics, requiring labeling with biotin for 16–18 h or even longer for in vivo labeling to produce sufficient biotinylated material for proteomic analysis.

The primary objective of biotin ligase engineering has been the development of enzymes with faster labeling kinetics and smaller size. These efforts have resulted into numerous modified enzymes including BioID2 (Kim et al., 2016), TurboID (Branon et al., 2018), miniTurboID, AirID (Kido et al., 2020), UltraID (Kubitz et al., 2022), and MircroID2 (Johnson et al., 2022). BioID2, UltraID, and MircoIDare smaller biotin ligases derived from *Aquifex aeolicus*, with the distinction that UltraID and MircoID have much faster labeling activities compared to BioID2. TurboID and miniTurbo were directly evolved from BioID with super biotinylation kinetics. TurboID has the size of BioID and is the most active enzyme to all known BirA ligases described to date supporting labeling kinetics down to 10 min or less. This enhanced activity, however, comes at the cost of high background labeling, which can be caused by stochastically biotinylated proteins in the same subcellular localization as the bait protein (May et al., 2020). The miniTurbo is smaller than TurboID and does not show high background activity. However, miniTurbo has about half the activity of TurboID and it exhibits biotin‐dependent stability. AirID is an engineered ancestral BirA with higher biotinoyl‐5‐AMP formation under low ATP conditions and is thus less toxic than TurboID. Compared with TurboID, AirID, UltraID, and MicroID system showed more specific tagging of interaction partners over long incubation periods, providing an additional useful PL tool for long‐lasting experiments in live cells (Johnson et al., 2022, Kido et al., 2020, Kubitz et al., 2022). Recently TurboID has been integrated with light‐sensitive Light‐Oxygen‐Voltage domain (LOV) to generate an engineered enzyme which can be precisely controlled by light (Lee & Cheah, Zhao, et al., 2023). This innovative approach ensures that LOV‐Turbo remains minimally active in the absence of light, virtually eliminating background activity. Upon exposure to low‐power blue light, however, LOV‐Turbo is swiftly activated. This capability not only facilitates precise temporal control over the protein labeling process but also significantly diminishes nonspecific background labeling, enhancing the specificity and accuracy of protein studies.

In addition to its extensive use in cultured mammalian cells, biotin ligases‐based PL has been integrated with other cutting‐edge procedures to be applied in live animals. The cardiac dyad proteome in live cardiomyocytes was characterized by knock‐in BioID2 into the GOI locus using the CRISPR‐Cas9 technology (Liu et al., 2021). By recombining adeno‐associated viruses (AAVs) to express TurboID driven by cell‐type‐specific promoters, researchers were able to track secretory proteins in blood plasma (Kim et al., 2021) and generate a secretome atlas of mouse (Wei et al., 2021).

### 4.2 Engineered ascorbate peroxidase (APEX) based PL

APEX is an engineered ascorbate peroxidase derived from pea or soybean (Martell et al., 2012). Peroxidase‐based proximity biotinylation proceeds via a different mechanism compared to biotin ligase‐based approaches. Cells expressing APEX fused POI are first incubated with a phenol derivative of biotin (biotin‐phenol). With the addition of H_2_O_2_, APEX can convert biotin‐phenol into biotin‐phenoxyl radical, which has a half‐life of <1 ms and can label electron‐rich amino acid residues (Tyr, Trp, His, and Cys) of proteins in the vicinity of ~20 nm of APEX (Rhee et al., 2013). A key advantage of APEX is that it permits labeling times as short as 1 min. The high activity of APEX makes it suitable for studying time‐resolved PPIs and transient PPIs in a fast signaling turnover. A disadvantage of APEX is that biotinylation requires H_2_O_2_ treatment of cells, which could potentially affect redox‐sensitive proteins or pathways, perturbing cellular processes, and cause cellular stress (Lennicke et al., 2015). APEX2 is an evolved mutant of soybean ascorbate peroxidase (Lam et al., 2015). Compared with APEX, APEX2 exhibits enhanced enzymatic efficiency and labeling precision in proteomic studies, enabling effective tagging of proteins across a broad abundance spectrum. Additionally, APEX2 is much more resistant to H_2_O_2_‐induced inhibition than APEX. However, APEX2's strong labeling activity could prevent the fine spatial resolution that is needed to determine the interactome of a POI. Since biotin‐phenoxyl radicals are not membrane‐permeable, both APEX and APEX2 are preferable to be used for proteomic profiling of membrane‐enclosed subcellular compartments, such as the mitochondria (Hung et al., 2017) and autophagosomes (Le Guerroué et al., 2017). Apart from the classic APEX2 substrate biotin‐phenol, alternative substrates with differing activities, such as biotin‐aniline, biotin‐naphthylamine (Zhou et al., 2019), and alkyne‐phenol (Li et al., 2020), are also available. However, their use in various subcellular conditions needs to be investigated further.

### 4.3 Horseradish peroxidase (HRP) based PL

HRP is another peroxidase that, when activated by H_2_O_2_, can convert a substrate into a highly reactive radical that in turn covalently tags neighboring proteins on electron‐rich amino acids. HRP can be used to biotinylate proximal proteins either by expressing the protein fused to HRP (Miyagawa‐Yamaguchi et al., 2014), or targeting the protein with a specific HRP‐conjugated antibody. Selective proteomic proximity labeling assay using tyramide (SPPLAT) (Li et al., 2014) is a method which utilizes the reaction between HRP and tryamide. A HRP conjugated antibody that recognizes the bait protein is added exogenously to cells. Following brief incubation with biotin‐tyramide (biotin‐phenol) and H_2_O_2_, HRP catalyzes the oxidation of tyramides into reactive free radicals, enabling proteins in the proximity of the bait to be biotinylated. Biotinylated proteins are then isolated by incubation with streptavidin‐agarose for MS analysis.

This approach has also been extended to be used on tissue samples, and HRP‐based biotinylation by antibody recognition (BAR) (Bar et al., 2018) has been developed. In a BAR system, the POI in a fixed and permeabilized tissue is initially recognized by the primary antibody. A secondary HRP‐conjugated antibody is then used to produce free radicals using biotin‐phenol and H_2_O_2_. The sample is lysed to enrich biotinylated proteins that are subjected to MS analysis. This approach avoids potential negative impacts of the additional tag on the POI activity. Moreover, antibody‐based detection prevents any artifacts related to protein overexpression and is especially useful in clinical applications for disease pathogenesis. However, highly specific monoclonal antibodies that are not sensitive to fixation artifacts are required for the application. Additionally, HRP‐based labeling methods are limited by the sensitivity of HRP to denaturation in certain conditions, restricting its application across various cellular compartments. While most cellular environments are neutral, the enzyme's stability can be compromised in specific contexts or treatments that induce acidic conditions, affecting its utility in the cytosol, nucleus, and mitochondria (Santos‐Barriopedro et al., 2021).

### 4.4 Ubiquitin‐like NEDD8 molecule‐based PL

The NEDDylator (Zhuang et al., 2013) system depends on fusing of the NEDD8 E2 enzyme (Ubc12) to the target E3 ligase's substrate‐binding domain. Endogenous E3 ligase substrates can be artificially NEDDylated using this arrangement. The substrate HB‐NEDD8 (His‐biotin tagged NEDD8) can only be transferred to the prey protein through direct contact with the bait protein. Prey tagged with NEDD8 can be enriched by affinity matrix and identified by MS. Direct bait prey contact is essential for NEDD8 labeling. This distinguishing feature of the NEDDylator system greatly reduces false positives. However, this system requires the endogenous NEDD8 pathway (NEDD8 E1‐activating enzyme, E2‐conjugating enzymes, and E3 ligases), and can interfere with the activities of related endogenous proteins. Additionally, the POI needs to be verified without NEDDylation activity before the application (Chu et al., 2020).

### 4.5 Pup ligase (PafA)‐based PL

PUP‐IT (pupylation‐based interaction tagging) system (Liu, Zheng, et al., 2018) utilizes the *Corynebacterium glutamicum* PafA as a tag fused with the bait protein, and its substrate Pup in the active form (PupE) with the C‐terminus glutamine was mutated to glutamic acid to be fused with other affinity tags for the following enrichment. For example, biotin‐containing BCCP‐fused Pup(E) allows the isolation of PupE‐conjugated protein using streptavidin beads. Unlike BioID or APEX, PafA binds the activated Pup and does not allow Pup to freely diffuse from itself, thus, restricting the labeling radius only to the proteins that interact with the bait protein. Because PafA and PupE are both proteins, a POI can be fused with PupE instead of PafA. In PUP‐IT2 (Yue et al., 2022), BCCP‐PupE is fused to a bait protein. PafA mediates the ATP‐dependent activation of PupE C‐terminal glutamic acid and the further ligation of PupE C terminus to the side chain of lysine on the prey protein. Thus, the bait and interacting partners will be linked covalently via the PupE protein. Functionally, PUP‐IT2 is comparable to PUP‐IT in its ability to label proximal proteins in vitro and cells but with less labeling background.

### 4.6 Photoactivated‐based PL

Singlet oxygen (^1^O_2_) that can be produced by light irradiation of a photocatalyst, is a highly reactive oxygen species with potential diffusion distance of ∼10 nm (Sies & Menck, 1992). It has been observed to oxidize methionine, tyrosine, histidine, and tryptophan promiscuously. Only specific amino acid residues (methionine, tyrosine, histidine, and tryptophan) have been found to undergo oxidation by ^1^O_2_. MiniSOG (mini Singlet Oxygen Generator) is a flavin‐binding protein that generates ^1^O_2_ under exposure to blue light (Nakane et al., 2021).

The photoactivation‐dependent PL (PDPL) platform (Zhai et al., 2022) uses blue light to illuminate a photosensitizer miniSOG‐fused POI and trigger singlet oxygen generation to oxidize proximal residues. These oxidized intermediates are then modified in living cells using an amine‐containing chemical probe. Compared to TurboID, PDPL provides more specific and comprehensive proteome coverage. Moreover, the miniSOG can be converted to a new form, singlet oxygen photosensitizing protein 3 (SOPP3), to achieve greater labeling efficiency (Mogensen et al., 2022).

### 4.7 The NeissLock proximity ligation

The NeissLock (Scheu et al., 2021) was established based on a secretory protein FrpC from *Neisseria meningitidis*. FrpC contains a self‐processing module (SPM), which displays calcium‐dependent autoproteolytic activity at an aspartate‐proline dipeptide bond. SPM can be used as a tag to fuse with the POI. The addition of calcium promotes conformational change in SPM to mediate FrpC auto proteolysis processing. Autoproteolysis follows protonation of the main‐chain nitrogen of proline, leading to formation of the anhydride, thus leading to the formation of a covalent bond between the binding protein and the POI. NeissLock system has been validated on three bait protein ODC, AZI, and EGFR to detect their protein interactions. Further work is still needed to investigate NeissLock in different cellular locations for different size ranges of protein complexes.

### 4.8 Split‐PL enzymes

Many PL enzymes, including HRP, APEX2, BioID, and TurboID, can be split into split‐forms to increase spatial specificity. In the split PL system, the PL enzyme is split into two inactive halves that are tagged with two POIs separately. When two POIs interact, the PL fragments can reconstitute into a functioning entity. The split‐PL technique is beneficial for research that needs greater spatial precision, such as characterizing protein complexes and membrane contact locations.

The BioID biotin ligase can be split at multiple points (De Munter et al., 2017; Kwak et al., 2020; Schopp et al., 2017). Split‐BioID yields substantially less background biotinylation than BioID since only proteins that assemble around this pair of interacting proteins are labeled. In contrast to split HRP (Martell et al., 2016) and split‐APEX2 (Han et al., 2019), split‐BioID and split‐TurboID (Cho et al., 2020) do not require the addition of cofactors or co‐oxidants.

Additionally, split‐TurboID labels faster than split‐BioID and identifies a more balanced set of proteomes. It's important to note that the strong binding affinity observed between POI pairs might also result from the overexpression of both tagged POIs (Han et al., 2019).

### 4.9 PL beyond PPIs

In addition to PPIs, PL techniques have been utilized to investigate protein–DNA (DamID), protein–RNA (APEX‐Seq), and cell–cells interactions (FucoID, EXCELL).

DamID (Orian et al., 2009) uses *E. coli* DNA adenine methyltransferase (Dam) fused to a POI. Expression of this fusion protein in vivo leads to preferential methylation of adenines in DNA surrounding the native binding sites of the POI. The adenine‐methylated DNA fragments can be isolated and identified by microarray approach.

Since APEX2 can directly biotinylate guanosine in RNAs within a few nanometers, APEX‐Seq (Fazal et al., 2019) utilizes PL to label and purify RNA for sequencing. APEX‐seq can provide RNA sequence information down to single‐nucleotide precision.

FucoID (Liu, Li, et al., 2020) is based on an interaction‐dependent fucosyl‐biotinylation for isolation of the endogenous tumor antigen‐specific T cells. On the surface of dendritic cells (DCs), *Helicobacter pylori a*(1,3) fucosyltransferase (FT) was implanted, when specific T cells came into contact with DCs, FTs marked the LacNAcylated glycans on the surface of the T cells with GDP‐fucose‐biotin. Labeled cells were sorted with flow cytometry and characterized by MS (Qiu et al., 2022). This approach has the potential to be translated to a clinical setting for cancer detection and stratify/optimize personalized treatment regimes. Similarly, an enzyme‐mediated proximity cell labeling technique (EXCELL) (Ge et al., 2019) for detecting cell‐cell contacts was developed, which relied on *Staphylococcus aureus transpeptidase Sortase A* variant (mgSrtA) as the labeling enzyme and a biotin‐tagged short peptide (biotin‐LPETG) as substrate. When mgSrtA is expressed on the surface of a target cell, it labels adjacent cells in the presence of biotin‐LPETG, enabling recording of cell–cell interactions.

## 5 COMPLEMENTARY AP AND PL MS

While AP results in the identification of stable protein complexes, PL enables the identification of proximity and transiently interacting proteins, which results in overlapping yet distinct PPI identifications. By integrating AP‐ and PL‐MS data, one can comprehensively characterize a protein's molecular context. However, the generation of individual cloning constructs, cell lines and the application of different protein purification protocols for each workflow are very laborious and time‐consuming. Several labs have been working on the integration of both approaches for sample preparation and protein purification to simplify the experimental procedures, such as FLAG‐BirA* tag (Hesketh et al., 2017; Lambert et al., 2015), and Multiple Approaches Combined (MAC)‐tag system (Liu, Salokas, et al., 2018).

The application of FLAG‐BirA*tag (Lambert et al., 2015) and later developed FLAG‐BioID2 (Aprosoff et al., 2023) shortens the time required for cloning and cell line generation. But different binding matrixes for AP and PL purification make it difficult in the following data integration process. MAC‐tag system combines the Strep‐tagII for AP and the BirA* for PL into a single construct (Figure 1D). Therefore, one construct and the same streptavidin matrix can be used for both AP and PL. With continued efforts, the MAC‐tag system has recently incorporated UltraID to create the MAC3‐tag (strep&UltraID) system (Liu et al., 2023; Salokas et al., 2022). Compared with the MAC‐tag, the MAC3‐tag is smaller in size and adds the ability to detect subtle changes or transient interactions over a short period. However, a unified protocol for both AP and PL conditions should take into consideration that proximity enzymes may use the trace amounts of biotin present in normal cell culture conditions, primarily sourced from fetal bovine serum (FBS). These biotin traces can result in nonspecific biotinylation and subsequent purification on a streptavidin matrix in the AP workflow, leading to incorrectly annotating direct binding partners of the bait protein. This disadvantage can be overcome by using the biotin‐free culture medium (May et al., 2020).

## 6 PROTEIN QUANTIFICATION BY MS

High‐performance mass spectrometry‐based bottom‐up proteomics offers in‐depth analysis of proteins in samples of complex mixtures, by identifying and quantifying corresponding peptides (Figure 1E). The two most commonly used proteomics approaches are data‐dependent acquisition (DDA) (Liu et al., 2004) and data‐independent acquisition (DIA) (Venable et al., 2004).

DDA methods have been extensively utilized in the proteomics field since their inception. The “top N” method is a widely used DDA experiment that selects the N most abundant precursor ions for fragmentation, which when combined with dynamic exclusion (DE), can increase precursor coverage by avoiding the repetitive collection of the most abundant precursor ions. To further boost the sensitivity of DDA for interactome investigations, an efficient data collecting approach without DE was recently developed (Zhang et al., 2022). In DDA investigations, peptide identification is typically achieved by searching fragment spectra against a protein sequence database. MaxQuant (Tyanova et al., 2016) and MSfragger (Kong et al., 2017) are currently two of the most extensively used programs for DDA data analysis.

Alternatively, methods based on DIA have been implemented to alleviate the limitations of DDA (Lambert et al., 2013), enabling the measurement of all peptides within a specified *m*/*z* window, rather than just the most intense ones. As a result, the DIA approach constantly fragments the same subset of peptides, resulting in improved repeatability across samples and quantitative accuracy compared to traditional DDA methods.

However, the size and number of predefined windows can significantly affect the sensitivity and specificity of the DIA experiment. Therefore, preliminary DDA data is often required to optimize window setup by identifying the *m*/z range where peptides are predominantly to be found, thus allowing for a more targeted and efficient DIA analysis (Lou & Shui, 2024). Moreover, due to the multiplexing nature of DIA MS2 spectra, DIA data processing demands substantial computational resources. Unlike DDA, where robust protein sequence database search tools and target/decoy strategies are standard, DIA quantification typically depends on either a spectral library or a protein sequence database, with a comprehensive spectrum library being crucial for alignment with the DIA data of interest, often constructed from in‐depth DDA data generated by offline‐fractionation of complex samples. The most extensively utilized software programs for the computational analysis of DIA data sets are OpenSWATH (Röst et al., 2014), Spectronaut (Bruderer et al., 2017), Skyline (Egertson et al., 2015), and DIA‐NN (Demichev et al., 2020). Recently, various methods for direct identification (without the need of a spectrum library) and spectrum prediction further enhance DIA data analysis (Gessulat et al., 2019; Tiwary et al., 2019). DIA‐NN (Demichev et al., 2020), for example, employs deep neural networks to distinguish real signals from noise in complex DIA data sets. It contains an extremely powerful library‐free module, that outperforms using a high‐quality spectrum library for some types of experiments.

With ongoing improvements in software and algorithms, DDA and DIA are expected to converge into a unified approach, Data Dependent‐Independent Acquisition (DDIA) (Guan et al., 2020), combining the strengths of both methods within a single analytical cycle. This hybrid approach allows for a quick acquisition of high‐quality DDA scans while dedicating most of the cycle time to DIA scans. The DDA scans are identified using conventional database search methods, and the identified peptides from these scans provide key information, such as retention time calibration and features for DIA extraction FDR control. The DDIA method has shown promise in increasing the identification of protein groups significantly.

In interaction proteomics, peptide signals can be recorded in both intact (MS1) and fragmented (MS2) form. While DDA has only the MS1 for quantification, due to its selective nature in fragment ion spectra acquisition. DIA acquires MS1 and MS2 for all flyable and fragmentable ions. Therefore, quantitative variation is significantly lower in DIA than DDA, with DIA showing a significant reduced coefficient of variation (CV) across replicates. This has been observed at both the peptide and protein levels, indicating that MS2‐based quantification of DIA data offers a more consistent and reliable measurement compared to MS1 quantification of DDA data (Fernández‐Costa et al., 2020).

DIA quantification in proteomics has evolved significantly over time, shifting from an initial reliance on MS2 data to sophisticated methodologies that integrate both MS1 and MS2 data (Huang et al., 2020), enabling more accurate and comprehensive analysis of complex proteomes. This evolution reflects broader trends in proteomics towards more integrative and data‐rich analytical approaches. Recent advances in MS instrumentation have also resulted in significant progress in interactomics. For example, the timsTOF mass spectrometer (Bruker) combines trapped ion mobility separation with TOF mass analysis. With parallel accumulation serial fragmentation (PASEF) (Meier et al., 2018) coupled with DDA, the timsTOF technology can achieve up to a fourfold increase in protein identification when compared to typical DDA analysis (Meier et al., 2021). Additionally, the DIA‐PASEF method outperforms DDA‐PASEF by at least 20% in terms of protein identification, underscoring the technological advancements and their impact on proteomic research (Huang et al., 2022). Furthermore, the Orbitrap Astral mass spectrometer (Thermo) integrates a mass‐resolving quadrupole with the Orbitrap, and an innovative Asymmetric Track Lossless (Astral) analyzer (Stewart et al., 2023). This state‐of‐the‐art instrument yields a proteome coverage of up to 5200 protein groups from a single mammalian cell (Bubis et al., 2024), demonstrating the potential in practical protein interactome from single‐cell insight. These technological breakthroughs not only enhance our understanding of complex biological systems but also pave the way for new discoveries in protein interactions and functions.

## 7 INTERACTOMICS DATA ANALYSIS

Protein interactomics research frequently results in a list of potential interactors that still contain unspecific “background” contamination. Contaminant interactions might be caused by the experimental environment. For example, proteins (usually high abundance proteins) that nonspecifically interact with the purification matrix, and proteins that never encounter each other within the living cell have significant affinity in the cell lysate. Furthermore, the use of the PL tag, with its increased labeling radius and activities, results in a higher background due to bystander protein labeling. A study of nuclear pore protein fused to BioID or TurboID, found that while both approaches can detect the nuclear pore complex identically, the overall number of proteins identified in the TurboID samples was ~2.7‐fold larger, including proteins present in other subcellular organelles (May et al., 2020).

These background interactions caused by reagents and sample processing are unavoidable. Being able to differentiate *bona fide* PPI from artifactual interactions is a challenge. This can be achieved through careful experimental design and stringent data analysis.

### 7.1 Experimental design and sample preparation

The most common way to remove background noise interactions from quantitative proteomics data is to employ negative control though the sample preparation. The choice of control samples has a significant influence on high confidence interaction (HCI) coverage and accuracy. The ideal negative control should be a tag fused inactive form of the POI that is unable to bind to its known interacting partners. However, in most cases, GFP is recommended as a negative‐control bait since it is not expected to have specific interactions in most organisms. Moreover, compartment‐specific controls, such as control with a nuclear localization sequence, control with a myristoylated membrane targeting sequence, or control with a nuclear export sequence, may be useful in identifying and filtering out nonspecific background proteins.

During the purification of samples, employing rigorous washing steps can help remove nonspecific bound proteins. This can be achieved by enhancing the salt concentration or modifying the composition of the wash buffer to increase stringency. However, it's important to note that such methods might also inadvertently eliminate genuine interacting proteins. Furthermore, quantitative techniques such as Stable Isotope Labeling by/with Amino Acids in Cell Culture (SILAC), Tandem Mass Tags (TMT), and Isobaric Tags for Relative and Absolute Quantitation (iTRAQ) (Chen et al., 2021) offer a means for the quantitative comparison of samples and controls. These methods play a crucial role in accurately identifying specific interactors.

### 7.2 Data analysis strategies

Various approaches range from knowledge‐based approach, where contaminants are manually identified and removed based on known attributes and prevalence, to more sophisticated probabilistic scoring methods that statistically differentiate true interactions from background noise.

The Contaminant Repository for Affinity Purification (CRAPome) (Mellacheruvu et al., 2013), is an online database to help researchers in pinpointing nonspecific or contaminant proteins that are commonly found in protein purification. Interaction data can be filtered by extracting information from the CRAPome, which encompasses 716 negative controls. Specifically, proteins that appear with a high frequency of detection across the CRAPome database are typically deemed unsuitable for subsequent validation efforts, thereby streamlining the identification of true PPIs.

Specific interaction partners are usually significantly more abundant in a test sample than in a control sample and may thus be used to score the interactions. Several computational algorithms have been developed to assign probabilities to PPIs (Figure 1F). These scoring methods utilize the quantitative features of MS‐based proteomics and consider negative controls, frequency of detection, and experimental reproducibility.

For example, the Normalized Spectral Abundance Factor (NSAF) (Paoletti et al., 2006) is given by dividing the spectral count of a protein by its length, and then normalizing this value across the experiment by dividing it by the sum of spectral count for all detected proteins. NSAF offers a normalized and comparably accurate way to assess protein levels across different samples or experimental conditions, but it is dependent on the detectability of peptides, which can be influenced by the chemical properties of peptides and the experimental setup.

Similarly, the Comparative Proteomic Analysis Software Suite (CompPASS) method (Sowa et al., 2009) uses *Z*‐scores and weighted *D*‐scores (WD‐scores) to differentiate true interactors from background proteins. *Z*‐scores are based on how often a protein appears with the bait across experiments, helping spot proteins that bind to the bait more often than would be expected by chance. WD‐scores adjust for how frequently a protein is found in all purifications and in controls, emphasizing interactions that are specific to a given bait and reproducibly observed across multiple experiments.

The Significance Analysis of Interactome express (SAINTexpress) method (Mellacheruvu et al., 2013) further builds on this framework by estimating the likelihood of PPIs through the integration of spectral counts with label‐free quantification data. It incorporates both direct spectral evidence and comparative abundance measurements and employs a Bayesian approach to evaluate the significance of observed interactions, allowing for the direct calculation of FDRs.

There are approaches that have been specifically developed to model and quantify the prevalent errors within PPI data sets. These strategies significantly reinforce the interpretation and utilization of PPI data sets.

Among these approaches, a directed graph model tailored for bait‐to‐prey systems, alongside a multinomial error model (Chiang et al., 2007), has been introduced to assess and characterize errors within PPI data sets. This method focuses on distinguishing false positives, false negatives, and evaluating the stochastic error rates that impact the overall data quality. Such an analytical framework is indispensable for refining the accuracy of PPI networks and enhancing the reliability of subsequent biological interpretations.

Furthermore, the Decontaminator algorithm (Lavallée‐Adam et al., 2011) assesses confidence by building a model of contaminants using a small number of representative control experiments. It compares the Mascot score of a putative prey against those observed in control experiments to assign a p‐value and FDR. This process not only improves accuracy in PPI identification but also reduces the need for control experiments. Decontaminator is easily incorporated into existing analysis pipelines and shown to identify contaminants more effectively than previously used approaches.

Additionally, aspects like the structural connections among direct physical interactions as well as co‐complex interactions, and Gene Ontology (GO) enrichment, network‐based with indirect association removal have been used to highlight likely genuine interacting partners. More recently, AI‐based structure prediction tool kit, AlphaPulldown (Yu et al., 2022), is specifically designed to utilize the power of AlphaFold‐Multimer for PPI screens and modeling of complex, higher‐order protein oligomers. This integration signifies a substantial advancement in PPI research, combining traditional experimental and computational analysis methods with AI technology to refine the identification and validation of PPIs.

## 8 GLOBAL PPI PROFILING

**Figure 2:**
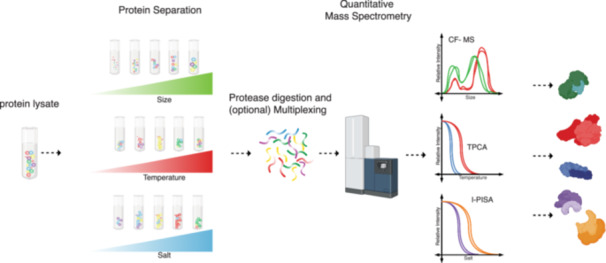
Methods for global protein–protein interactions profiling. Proteins are separated by size, temperature, salt, or another characteristic to group proteins that share such characteristics and predict those likely to form complexes. Proteins are digested and subjected to quantitative mass spectrometry, often accomplished by multiplexing with tandem mass tag labeling to increase throughput and minimize missing values. Protein denaturation/aggregation curves are generated from the relative intensities of protein detected in each fraction. Protein complexes are inferred from a deconvolution of these data, where proteins with similar curves are predicted to form complexes. [Color figure can be viewed at wileyonlinelibrary.com]

In parallel with the methods described above, which focused on characterizing interactions of specific POI, efforts have also been placed on developing methods that capture PPI networks at a global scale, that is, at the whole‐cell level. Indeed, global protein interactomes, as well as their dynamics in response to stimuli, provide signatures of cellular states (Vidal et al., 2011). Hence, such methods would allow probing questions pertaining to the coordination of cellular pathways and signaling cascades. Recent method developments have enabled high‐throughput and efficient characterization of global PPI networks. These global PPI profiling techniques take advantage of the observation that proteins that interact and form complexes tend to stabilize each other, thereby sharing certain biochemical properties, such as size or solubility. PPIs are fractionated as a function of these biochemical properties, and subsequently quantified by MS. Proteins with similar abundances across fractions are predicted to interact. Currently, three MS‐based global PPI profiling methods have been developed, which include Co‐fractionation (CF), Thermal Proximity Coaggregation (TPCA), and Ion‐Based Proteome‐Integrated Solubility Alteration (I‐PISA) (Figure 2) (Beusch et al., 2022; Kristensen et al., 2012; Savitski et al., 2014; Tan et al., 2018). CF‐MS fractionates proteins using liquid chromatography, most commonly by size exclusion (SEC), also known as gel filtration. In this technique, interacting proteins/complexes are separated into fractions by their differences in size, and individual proteins are quantified by MS (Havugimana et al., 2012; Kristensen et al., 2012). Proteins with highly correlated elution profiles are predicted as likely interacting partners. A second approach, TPCA‐MS, is performed by exposing intact cells or tissues to increasing temperatures, thereby inducing protein denaturation and precipitation (Savitski et al., 2014; Tan et al., 2018). Proteins in complex are expected to provide thermal stability to each other and to exhibit similar solubilities under the same cellular conditions. Proteins are quantified from the soluble fractions, usually by multiplexed mass spectrometry using isobaric tags, thus providing a readout of denaturation/aggregation curves for each protein. The similarity between thermal stability curves is then used to predict protein associations. A more recently developed method, I‐PISA, is a similar curve‐based method as TPCA, but uses increasing salt concentrations to impact protein solubility. An advantage of TPCA and I‐PISA is that heat or salt‐induced denaturation are often performed before cell lysis, which may prevent proteins in different subcellular compartments from interacting before the fractionation step (Richards et al., 2021).

**Table 3:** Overview on the commonly used protein interaction databases.

Database	Website	Source of data collection	Approx. number of PPIs	Usage	Unique features
HuRI	www.interactome-atlas.org	Experimentally acquired from scientific publications	Interactors: 9094 Binary Interactions: 64,006	Storing and managing experimentally acquired PPI data	Focuses specifically on human protein interactions, providing a comprehensive map of the human interactome.
IntAct	https://www.ebi.ac.uk/intact/	Manual curation and automated scraping methods from publications	Interactors: 134,556 Interactions: 808,073 Binary Interactions: 1,293,508	Access and analyze protein interaction data	Part of the EMBL‐EBI suite of databases, known for its detailed curation and integration with other EBI resources.
HIPPIE	http://cbdm-01.zdv.uni-mainz.de/~mschaefer/hippie/	Aggregates data from multiple sources	Not specified	Enhancing data set robustness by including verified interactions	Offers a score system for interaction confidence and integrates various sources to provide a comprehensive view of PPIs.
STRING	https://string-db.org/	In silico predictions, knowledge transfer, experimental data	Interactors: 59.3 million Interactions: >20 billion	Comprehensive database for exploring known and predicted PPIs	Known for its extensive data set and for providing predicted interactions along with known ones, and functional enrichment analysis.
BioGRID	https://thebiogrid.org/	Experimentally acquired data, automated scraping methods	Interactions: 2,134,950 PTM sites: 563,757	Access and analyze protein interaction data	Include interactions, chemical associations, and posttranslational modifications (PTM) from 84,441 publications.
GeneMANIA	http://genemania.org/	*In silico* prediction, data from multiple sources	Interactors: 166,691 Binary Interactions: 660,554,667	Supporting functional enrichment analysis using gene ontology	Unique for its ability to generate hypothesis about gene function, gene‐gene interactions, and to visualize interaction networks.

The development of global PPI techniques has accelerated our understanding of the systems‐level response of PPI dynamics to intrinsic and extrinsic factors. For example, in work from Mateus et al., high‐throughput TPCA was employed in over 100 *E. coli* strains, each containing single‐gene deletion mutants (Mateus et al., 2020). Notable among several findings was that the thermal landscape of *E. coli* could be used as a readout of enzymatic activity in vivo, and therefore, to infer changes in patterns of metabolic pathway activity (Mateus et al., 2020). Global PPI profiling methods have also been employed to characterize the temporality and coordination of host‐pathogen interactions. Time‐course experiments using TPCA have elucidated how viruses, such as human cytomegalovirus (HCMV), herpes simplex virus type 1 (HSV‐1), severe acute respiratory syndrome coronavirus 2 (SARS‐CoV‐2), and Kaposi's sarcoma herpesvirus (KSHV) modulate host protein complexes over the course of infection (Hashimoto et al., 2020; Justice et al., 2021; Reed et al., 2024; Selkrig et al., 2021). Temporal interactions identified from these studies have driven the discovery of specific processes involved in either virus replication or host defense mechanisms, for example, crosstalk between viral DNA sensing and DNA damage response during HSV‐1 infection (Justice et al., 2021) and SARS‐CoV‐2‐driven changes in the thermal stability of certain host factors (Selkrig et al., 2021).

Accumulation of such global PPI data sets has also afforded the comparison of interactome alterations induced by different pathogens, leading to the identification of common strategies to modulate host cells among herpesviruses (Reed et al., 2024) or among jumbo bacteriophages (Fossati et al., 2023). Comparison of PPI networks predicted by global interactome profiling techniques may also be leveraged to study similarities among various related organisms or tissue types at the protein‐level. Analysis of multiple CF‐MS data sets have been used to identify tissue‐specific complexes in mice (Skinnider et al., 2021) and to construct a pan‐plant interactome (McWhite et al., 2020; Sun et al., 2023).

Global interaction methods have also allowed researchers to investigate the evolutionary conservation of protein complexes and networks, even among distantly related organisms. In this regard, CF‐MS has led the charge, providing experimental evidence for protein complex composition that spans nearly 1 billion years of evolution (Havugimana et al., 2012; Wan et al., 2015). CF‐MS experiments from multiple laboratories have continued to generate increasingly detailed evolutionary protein networks. Recently, these data sets have been leveraged by Skinnider and colleagues to assemble a unified CF database (CFdb) from meta‐analysis of 411 CF‐MS experiments, representing protein abundances, phosphorylations, and interactions of organisms across the tree of life (Skinnider et al., 2023). Complementing CF‐MS studies, an atlas of thermal stability profiles was assembled across 13 species (Jarzab et al., 2020). This “meltome” provided intriguing evidence for proteins that have maintained their stability throughout evolution, and for certain proteins, such as mitochondrial respiratory chain members, that likely have evolved to function at consistently higher melting temperatures.

Extracting reproducible and biologically meaningful insights from global interaction profiling data sets requires optimized analytical and computational pipelines. Analytically, the collection of multiple fractions per sample per replicate is required. Increasing the number of fractions can theoretically allow for better resolution of protein complexes or a protein's biochemical characteristics, which would lead to more accurate PPI predictions. Experiments have shown benefits in collecting up to 40 s fractions for CF‐MS (Skinnider et al., 2021). while 8–10 temperature fractions are typical for TPCA (Sun et al., 2023). Depending on the experimental design, the number of samples to be quantified by mass spectrometry can be substantial and time‐prohibitive. To circumvent these drawbacks, sample processing and workflow efficiency can be optimized (e.g., 96‐well FASP approach), while sample multiplexing approaches, such as tandem mass tagging (TMT), have been utilized for increased throughput (Havugimana et al., 2022; Tan et al., 2018). Additionally, Reed and colleagues have improved the throughput of TPCA by determining an optimum set of five melting temperatures, which produced at least the equivalent predictive performance as a typical 10 temperature melting strategy (Bludau et al., 2023; Reed et al., 2024).

From a computational perspective, deconvolution of the large data sets produced by global PPI profiling and accurate prediction of PPIs and complexes is nontrivial. Generally, analysis software compares co‐fractionation traces or melting curves assembled from protein abundances across fractions using statistical models or machine learning. Analysis pipelines developed for CF‐MS are powerful and boast accurate predictive capabilities by either utilizing Analysis pipelines developed for CF‐MS are powerful and boast accurate predictive capabilities by either utilizing Bayesian analysis on existing data such as PCprohet (Fossati et al., 2021) or perform a complex‐centric analysis like Ccprofiler (Heusel et al., 2019) or by applying machine learning on coelution data sets with a Gaussian model in PrInCE and random Forrest classifiers with EPIC (Hu et al., 2019; Stacey et al., 2017). TPCA workflows are challenging, as many proteins fall within a narrow distribution of curves. This underscores the importance of computational methods to differentiate between curves and provide adequate sensitivity and specificity for PPI prediction. commonly used computational methods for analyzing TPCA‐derived stability data involve generating log‐logistic curves from protein solubility data and comparing midpoint melting temperatures (∆*T*_m_) (Martinez Molina et al., 2013; Savitski et al., 2014) or calculating the Euclidian distance between the fitted curves (Tan et al., 2018). Recent algorithms have focused on Bayesian analysis (Fang et al., 2021) and other improvements have been made to the performance and accessibility of the existing algorithms (Ji et al., 2022; Kurzawa et al., 2021; Sun et al., 2023). A recently developed software, *Tapioca*, sought to further improve the prediction of de novo PPIs in dynamic contexts (Reed et al., 2024). *Tapioca* employs a logistic regression‐based ensemble machine learning framework to improve PPI prediction by integrating curve‐based data with auxiliary data, such as existing interaction data, known protein physical properties, domain relationships, and tissue‐specific functional networks (Reed et al., 2024).

Each individual global PPI profiling method has proven powerful and has been utilized to successfully predict PPI networks, yet each has their inherent strengths and biases. As most global PPI analyses to date have employed a singular global PPI profiling technique, there is an opportunity to have a richer perspective of protein networks through complementarity. Notably, since Tapioca's PPI predictions are agnostic to curve shape, its predictions are generalizable independent of the data type (CF‐MS, TPCA, I‐PISA, etc.) (Reed et al., 2024) Overall, future studies will benefit from the integration of heterogenous global PPI profiling data sets (Reed et al., 2024) and from the systematic evaluation of data collection and analysis pipelines (Pang et al., 2020; Salas et al., 2020).

## 9 VERIFICATION OF INTERACTIONS

It is important to note that a majority of identified interactions via high‐throughput proteomic approaches are not subjected to subsequent validation. However, it is commonly accepted that PPIs validated by multiple experimental methods, especially by an orthogonal technique or confirmed by different research teams are considered to be of high confidence (Reguly et al., 2006). For example, researchers utilized both high‐throughput yeast two‐hybrid (Y2H) experiments and AP‐MS approach to obtain a high‐quality SARS‐CoV‐2–human PPI network for drug repurposing study (Zhou et al., 2023). Besides Y2H, other frequently employed method for interaction validation is co‐immunoprecipitation (co‐IP). Co‐IP investigations can be performed in several ways in most lab conditions using cell lines or tissues with overexpressed or endogenous proteins. However, immunoblotting (e.g., western blot) can detect as low as 0.1 ng of POI with specific antibody. While conventional liquid chromatography–mass spectrometry has a lower detection limit (typically between ng/mL and pg/mL), it is significantly more sensitive than antibody‐based immunoassays. The insufficient sensitivity and measurement dynamic of co‐IP is also reflected in its inability to detect transient interaction with the current protocol configuration. Previous study (Liu et al., 2021) employed immunoblotting to verify about 190 interaction pairs detected by complementary AP‐ and PL‐MS. The validation experiments (dot blot) confirmed roughly 60% of the selected HCIs.

Any of the previously reviewed methods can also serve as approach to orthogonally validate the interactions revealed by the other techniques. For instance, in a system level analysis of human protein phosphatase interactome by AP‐MS (Yadav et al., 2017), 71% of chosen HCIs (333 pairs) could be confirmed by BioID, and 69% of selected HCIs (16 pairs) could be verified by co‐IP.

Another popular method to verify PPI in vivo is microscopy‐based image analysis, such as fluorescence resonance energy transfer (FRET) assay (Sekar & Periasamy, 2003), and bioluminescence resonance energy transfer (BRET) assay (Xu et al., 1999). FRET and BRET both work on the basis of nonradioactive energy transfer between a donor and an acceptor. Two POIs are expressed and subjected to the binding experiment, one with the donor and the other with the acceptor. The interaction of POIs may then be assessed using specific phenomena. Similarly, bimolecular fluorescence complementation (BiFC) (Kerppola, 2008) uses halved fluorescent protein to tag POIs. When POIs interact, deactivated fluorescent protein halves are brought together, restoring the fluorescence. For example, the split Venus‐based BiFC was employed to validate the novel interactors of cerebral dopamine neurotrophic factor (CDNF) discovered by AP‐MS (Eesmaa et al., 2022). These findings indicate that CDNF protects cultured sympathetic and dopamine neurons from ER stress‐induced apoptosis through regulating unfolded protein response signaling pathways.

Finally, to reach higher levels of molecular precision, high‐resolution technique analyses, surface plasmon resonance, X‐ray crystallography, nuclear magnetic resonance, and even hydrogen‐deuterium exchange MS can provide information on primary PPI affinities and dynamics of the interactions, whereas X‐ray crystallography, nuclear magnetic resonance, and cryo‐electron microscopy are recommended to refine structural information of proposed PPI.

## 10 PROTEIN INTERACTIONS DATABASES

The protein interactome databases are critical for storing and managing vast volumes of interactomics data. Most databases contain experimentally acquired PPI data from scientific publications through both manual curation and automated scraping methods (Table 3), for example, the Human Reference Protein Interactome Mapping Project (HuRI) (Luck et al., 2020), IntAct (del Toro et al., 2021) or aggregates data from multiple sources, enhancing the data set's robustness by including interactions verified through various experimental methods (e.g., HIPPIE, Alanis‐Lobato et al., 2017). A few databases, for example, Search Tool for the Retrieval of Interacting Genes/Proteins (STRING) (Szklarczyk et al., 2022), Biological General Repository for Interaction Datasets (BioGRID) (Oughtred et al., 2021), GeneMANIA (Zuberi, Franz, et al., 2013) also integrates data from in silico‐based prediction, and knowledge transfer between organisms. These databases provide a platform for researchers to access and analyze PPI data. STRING (Search Tool for the Retrieval of Interacting Genes/Proteins) is now the most comprehensive database in terms of the number of PPIs (>20 billion) among them (Bajpai et al., 2020; Szklarczyk et al., 2022). The STRING database can be accessed via the web interface to search for the known and predicted interaction partners of a POI. The STRING database, accessible through its web interface, enables users to explore both known and predicted interactions of POIs. It details the experimental methods used for data collection and often includes references to related scientific literature. Additionally, STRING supports functional enrichment analysis using gene ontology annotations, enhancing understanding of protein functions. The database is regularly updated to ensure current information, and users can download the data set in various formats for compatibility with different applications.

As no one source gives a full perspective of all interactions, it is highly recommended to combine multiple sources to construct a comprehensive PPI network for one's own research.

## 11 APPLICATION OF PPI NETWORK

The PPI network illustrates the interactions and associations of proteins across various cellular compartments, providing valuable information about the dynamic organization of the proteome within the cellular architecture.

Moreover, PPI network revels the molecular basis of cellular activities required for cell viability and homeostasis. As a result, aberrant PPIs have been implicated in a variety of conditions such as disease progression and virus infection., and therapeutic interventions.

The potential to modulate PPIs has been shown can offer a therapeutic paradigm for drug discovery. Therefore, comprehensive systems‐level mapping of PPIs will help discover new interaction‐based treatments while also advancing our knowledge of protein sociology and their physiological roles.

### 11.1 Blueprint of the human proteome

Blueprint of human proteome refers to the comprehensive mapping and characterization of all the proteins in the human body, including their interactions, functions, and modifications, which have been challenging to study at the systems level.

The BioPlex Network has made significant contributions through high‐throughput AP‐MS. BioPlex 1.0 (Huttlin et al., 2015) laid the foundation of the human protein‐protein interaction network by screening thousands of PPIs. The subsequent BioPlex 2.0 (Huttlin et al., 2017) expanded this interactome over 56,553 interactions from 5891 AP‐MS experiments. BioPlex 3.0 (Huttlin et al., 2021) further extended these efforts, revealing 118,162 interactions among 14,586 proteins in 293 T cells. Additionally, it provided another PPI network that is generated from 5522 immunoprecipitations in HCT116 cells. Both networks offer insights into the complex organization of the human cell proteome and how PPI varies across cell types to accommodate specific cellular functions.

PL and crosslinking add another essential layer by placing these interactions within the context of cellular architecture, revealing not just who is interaction partner but where these interactions occur within the cell. A prime example is the use of the APEX that enables the generation of unique biotinylation patterns, to develop the “organelle barcodes” system for accurately identifying the subcellular location of proteins (Lee et al., 2016). This approach is highly specialized in determining the protein localization within the endoplasmic reticulum, as well as distinguishing between different subcompartments within the mitochondria such as the matrix, intermembrane space (IMS), and outer mitochondrial membrane (OMM) (Lee et al., 2016). Similar, the development of a noninvasive technique utilizes poly (lactic‐co‐glycolic acid) (PLGA) nanoparticles, functionalized with dimethyldioctadecylammonium bromide (DDAB), to deliver protein cross‐linkers directly into mitochondria (Chen et al., 2023). This approach preserves the physiological relevance of observed interactions and structures of mitochondrial proteins without the organelle isolation and was able to identify 74 pairs of novel PPIs.

Besides mitochondria, the “MS‐microscopy” platform is a database that uses proteomes biotinylated by organellar markers to locate and quantify protein interactors, and assign the subcellular localization to the POI (Liu, Salokas, et al., 2018; Liu et al., 2020). This method is particularly effective at identifying alterations, which makes it a potent tool for investigating disease‐associated mutations that result in aberrant protein distribution and localization (Keskitalo, Haapaniemi, Einarsdottir, et al., 2019; Keskitalo, Haapaniemi, Glumoff, et al., 2019; Kondelin et al., 2018). In a remarkable feat of human cell proteome, researchers have created a detailed map of the human cell using BioID (Go et al., 2021), characterizing 192 subcellular markers. The “humancellmap” shows the interaction and subcellular localizations of more than 4000 distinct proteins in HEK293 cells, highlighting key proteins at the ER‐mitochondrial boundary crucial for regulating mitochondrial balance.

Complementing these methods, global PPI mapping systematically identifies the interactions between proteins across the entire set of proteins expressed by human cells. This technique is unbiased and comprehensive, making it the perfect approach towards creating a blueprint of the human proteome (Lundberg & Borner, 2019). For example, by combining MS‐based proteomics with sequential cell fractionation, researchers have developed a novel, high‐throughput workflow for mapping the dynamics of proteome and phospho‐proteome across subcellular compartments (Martinez‐Val et al., 2021). This method allows for the detailed analysis of spatial‐temporal regulation of EGFR signaling dynamics in different biological systems, including cell lines and mouse tissues (Martinez‐Val et al., 2021). However, the technical requirements for global proteome mapping are challenging. Most global PPIs profiling methods rely on some form of gradient separation to partially sort organelles. Achieving identical gradient fractions across multiple experiments is practically impossible (Gatto et al., 2010). Moreover, protein translocations are often partial, and many proteins exhibit complex subcellular patterns that involve shifts between subcellular localizations that are more quantitative than qualitative (Borner, 2020). The complex situations highlight the importance of careful thought in determining the definition of protein translocation by its interaction network.

In general, by integrating proteomics data with other omics resources, researchers can achieve a multidimensional understanding of protein functions and interactions within the complicate cellular landscape, driving forward our understanding of human health and disease.

### 11.2 Probing disease mechanisms

Understanding how genetic variations like disease‐associated mutations or alternative splicing and environmental factors like drug exposure, growth factors, or hormones affect dynamics of PPI interactome can help to explain protein complex assembly, regulation, cellular functions, and pathogenic processes.

**Figure 3:**
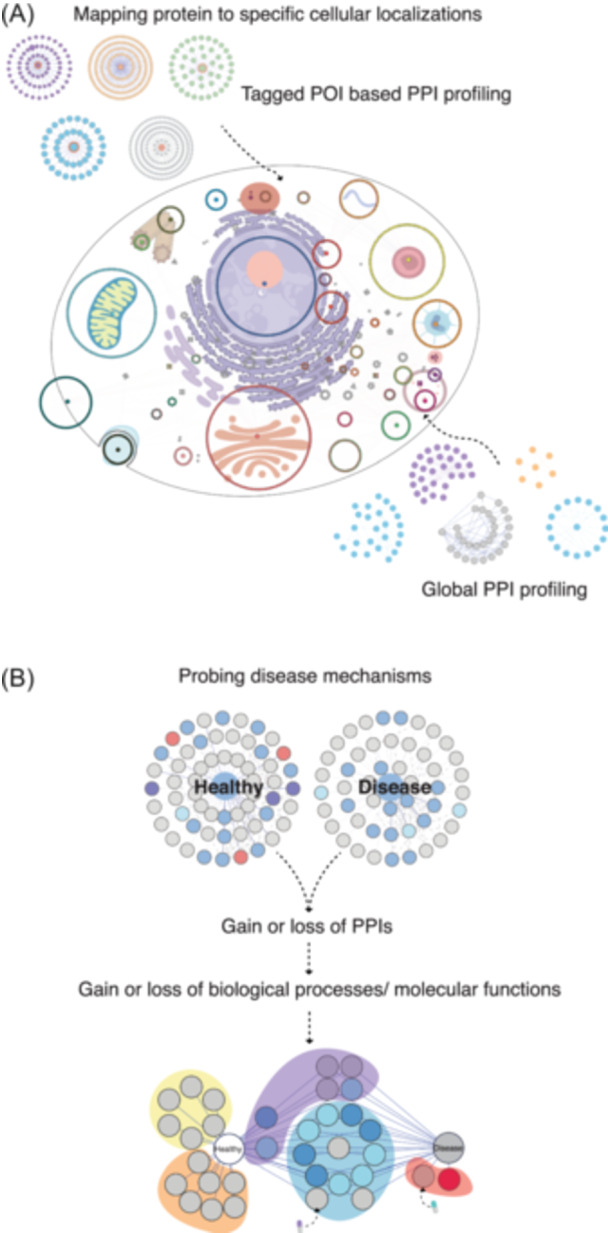
Application of protein interactome in the spatial proteomics and the exploration of disease mechanisms. (A) In protein network analysis, nodes represent individual proteins, and edges (the lines connecting the nodes) show the interactions between them. The interaction information obtained by tagged PPI profiling and global PPI profiling or XL‐MS can integrate with known protein subcellular information for protein localization predication and provides a detailed insight into the functional architecture of the cell. (B) The alterations in the network illustrated by changes in the connections (edges) to highlight biological pathways or molecular functions that are either lost from or gained in the disease state. The solid line represents the novel interaction, whereas the dashed line indicates the known interaction. These changes underpin the pathophysiology introduced by disease‐causing mutations. Using a network‐based strategy, drug target candidates can be further predict based on protein structural information. [Color figure can be viewed at wileyonlinelibrary.com]

It is feasible to compare disease states to the wild‐type interactome and identify how PPI networks evolve during disease pathogenesis by introducing mutant proteins or exogenously pathogen proteins (Gordon, Jang, et al., 2020; Liu et al., 2021; Richards et al., 2021). Changes in PPIs reflect the development of the genetic disease state, potentially bridging the gap between genotype and phenotype (Göös et al., 2019; Kaustio et al., 2017) (Figure 3B). For example, characterizing neurodegenerative disease has proven to be challenging, because the role of numerous neurodegenerative‐causing genes and the function of aggregation‐prone proteins are poorly understood. Utilizing AP‐MS to identify the interactomes of Alzheimer's disease (AD), Huntington's disease (HD), Parkinson's disease (PD), and spinocerebellar ataxia type 1 (SCA1) (Hosp et al., 2015), has achieved through direct comparisons between healthy and pathogenesis‐specific interactomes. Similarly, a study (Markmiller et al., 2018) used APEX‐tagged G3BP1, a stress granule, unveiled a vast array of proteins associated with stress granules in neuronal cells and discovered that amyotrophic lateral sclerosis (ALS)‐related mutations in HNRNPA2B1 were associated with an increased propensity to form stress granules.

Recently, AP‐MS was used in combination with artificial intelligence (AI)‐driven structural modeling and experimental validation to analysis 30 missense variants of the SLC6A8 gene and their impact on creatine deficiency syndrome (CDS) (Ferrada et al., 2024). The results confirmed the pathogenicity of 13 variants previously associated with CDS and identified six variants as benign and nine as deleterious (Ferrada et al., 2024). This comprehensive approach offers insights into the molecular basis of CDS and establishes a framework for assessing the effects of genetic variants on protein function.

Virus infections represent another critical factor that can significantly change the PPI landscape, as viruses often hijack the cellular machinery of host for replication and spread. By interfering with or co‐opting host protein complexes, viruses can induce profound changes in the assembly, regulation, and function of protein networks. This interaction not only facilitates the viral lifecycle but can also disrupt normal cellular processes, leading to disease manifestation. Therefore, exploring the effects of viral infections on the PPI interactome is essential for understanding virus–host interactions and developing therapeutic strategies against viral diseases. One of the great examples is the Krogan lab, which studies a broad spectrum of bacterial and viral pathogens, including but not limited to HIV (Jäger et al., 2011), SARS‐CoV‐2 (Bouhaddou et al., 2020; Gordon, Hiatt, et al., 2020; Gordon, Jang, et al., 2020), Influenza A Virus (IAV) (Haas et al., 2023), Hepatitis B and C Viruses (HBV, HCV) (Ramage et al., 2015), Dengue virus (De Maio et al., 2016), Zika virus (Shah et al., 2018), Ebola virus (Batra et al., 2018), Herpesvirus (Davis et al., 2015), Chlamydia (Mirrashidi et al., 2015), Pseudomonas (Inclan et al., 2016), and Tuberculosis (Penn et al., 2018). These projects seek to uncover the fundamental mechanisms of pathogenesis and identify numerous potential drug targets against different pathogens, representing a significant contribution to our understanding of infectious diseases. However, it should be noted that the prevalent approach in AP‐MS studies typically express individual viral proteins in host cells rather than the complete viral genome, lacking viral protein cooperation and potentially resulting in artificial interactions that do not occur during natural infection. Such overexpression artifacts can obscure the data interpretation and hinder the recognition of critical virus–host interactions. Nevertheless, the field adapts by adopting more comprehensive models that can more accurately mimic the complexity of viral infection. For examples, approaches that involve infecting cells with the whole virus or utilizing noninfectious viral replicons promise a more accurate reflection of the interplay between virus and host, thus providing deeper insight into their interactions.

The identification of crucial nodes (proteins) or edges (PPIs) within a disease/pathogen‐specific PPI network enables the discovery of novel pharmacological targets and the design of small molecules or peptides aimed at modulating these networks for therapeutic effects. Many computational tools, including network‐based methods, machine learning methods, and data integration methods, have been developed to find pharmacological targets in PPI networks (Figure 3B).

The topology and other properties of the PPI network, such as degree centrality, betweenness, and clustering coefficient, are examined in network‐based approaches to identify nodes that are critical for the PPI function (Figure 3B). An earlier study (Zhu et al., 2009) found that of the top 200 proteins ranked based on their topology in the human interaction network, 94 matched with known drug targets in DrugBank. Machine learning approaches, on the other hand, identify proteins based on the PPI network using a variety of algorithms such as decision trees, random forests, and support vector machines. For example, a deep learning‐based computational framework (Tsuji et al., 2021) was developed and applied on PPI networks to prioritize putative drug targets for Alzheimer's disease. The method was successful in identifying novel protein targets and inferring promising repositionable drugs for the disease. Furthermore, data integration methods usually combine PPI network resources with other omics data, such as epigenetics, genomics, and metabolomics data, to identify potential drug targets based on their relevance to a particular disease. By integrating information from PPI network and disease‐gene associations, researchers were able to systematically screen and prioritize disease indications for drug targets (Han et al., 2021). In addition to drug target identification, PPI networks can also be used to identify drug combinations that can modulate the network to achieve a desired therapeutic outcome. For example, PRODeepSyn (Wang, Zhu, et al., 2022) integrates the PPI network data with other omics data using the deep learning model graph convolutional network to predict anticancer synergistic drug combinations.

## 12 CONCLUDING REMARKS

Recent advances in MS‐based protein interactomics have significantly expanded our knowledge of how proteins interact within cells. These methods differ in sensitivity, specificity, and resolution for the study of PPIs in various biological contexts. Each approach has its own advantages and limitations. As the field progresses, there is a growing demand for an integrated workflow that combines these techniques to take advantage of the best aspects of each method, resulting in a more detailed protein interactome.

Nevertheless, the addition of tags, the generation of cell lines, labeling, and the immobilization of solid phases could become obsolete, and information on affinities, kinetics, and structure should be accessible via new, powerful, and effective analytical approaches. Techniques such as, global protein profile methods have been developed towards this direction, implying a shift towards more efficient and powerful analytical methods.

Another promising prospect is the development of ultra‐sensitive, microfluidics‐based interaction proteomics that can analyze PPIs at the single‐cell level (Furlan et al., 2019; Gebreyesus et al., 2022). These developments would reduce the amount of input material while enhancing the physiological relevance of the identified interactome.

Moreover, the field is poised for transformative developments that could monitor PPI real‐time dynamics (Thakur & Movileanu, 2019), in vivo interactome in complex tissues (Skinnider et al., 2021), and the integration of interactomics data with AI and machine learning models for predicting cellular behavior and drug responses (Ferrada et al., 2024; Li et al., 2022). These advancements are expected to enhance our understanding of the molecular basis of life and disease, opening an avenue for personalized medicine.

## AUTHOR CONTRIBUTIONS

**Xiaonan Liu**: Conceptualization; data curation; formal analysis; funding acquisition; investigation; methodology; project administration; resources; software; supervision; validation; visualization; writing—original draft; writing—review and editing. **Lawrence Abad**: Formal analysis; investigation; methodology; writing—original draft. **Lopamudra Chatterjee**: Data curation; formal analysis; methodology; writing—review and editing. **Ileana M. Cristea**: Data curation; formal analysis; funding acquisition; investigation; methodology; project administration; resources; supervision; validation; visualization; writing—original draft; writing—review and editing. **Markku Varjosalo**: Conceptualization; data curation; formal analysis; funding acquisition; investigation; methodology; project administration; resources; software; supervision; Validation; visualization; writing—original draft; writing—review and editing.
